# Accumulated hypertension burden on atrial fibrillation risk in diabetes mellitus: a nationwide population study

**DOI:** 10.1186/s12933-023-01736-4

**Published:** 2023-01-19

**Authors:** JungMin Choi, So‑Ryoung Lee, Eue‑Keun Choi, HuiJin Lee, MinJu Han, Hyo-Jeong Ahn, Soonil Kwon, Seung-Woo Lee, Kyung‑Do Han, Seil Oh, Gregory Y. H. Lip

**Affiliations:** 1 Division of Cardiology, Department of Internal Medicine, Seoul National University Hospital, Seoul, Republic of Korea; 2 Department of Internal Medicine, College of Medicine, Seoul National University, Seoul, Republic of Korea; 3 Department of Medical Statistics, College of Medicine, Catholic University of Korea, Seoul, Republic of Korea; 4 Statistics and Actuarial Science, Soongsil University, Seoul, Republic of Korea; 5 Liverpool Center for Cardiovascular Science, University of Liverpool and Liverpool Chest & Heart Hospital, Liverpool, UK; 6 Department of Clinical Medicine, Aalborg University, Aalborg, Denmark

**Keywords:** Atrial fibrillation, Type 1 diabetes, Type 2 diabetes, Hypertension, Cardiovascular complications

## Abstract

**Background:**

Patients with diabetes mellitus have an increased risk of incident atrial fibrillation (AF). The effect of accumulated hypertension burden is a less well-known modifiable risk factor. We explored the relationship between accumulated hypertension burden and incident AF in these patients.

**Methods:**

We evaluated data for 526,384 patients with diabetes who underwent three consecutive health examinations, between 2009 and 2012, from the Korean National Health Insurance Service. Hypertension burden was calculated by assigning points to each stage of hypertension in each health examination: 1 for stage 1 hypertension (systolic blood pressure [SBP] 130–139 mmHg; diastolic blood pressure [DBP] 80–89 mmHg); 2 for stage 2 (SBP 140–159 mmHg and DBP 90–99 mmHg); and 3 for stage 3 (SBP ≥ 160 mmHg or DBP ≥ 100 mmHg). Patients were categorized into 10 hypertensive burden groups (0–9). Groups 1–9 were then clustered into 1–3, 4–6, and 7–9.

**Results:**

During a mean follow-up duration of 6.7 ± 1.7 years, AF was newly diagnosed in 18,561 (3.5%) patients. Compared to patients with hypertension burden 0, those with burden 1 to 9 showed a progressively increasing risk of incident AF: 6%, 11%, 16%, 24%, 28%, 41%, 46%, 57%, and 67% respectively. Clusters 1–3, 4–6, and 7–9 showed increased risks by 10%, 26%, and 45%, respectively, when compared to a hypertension burden of 0.

**Conclusions:**

Accumulated hypertension burden was associated with an increased risk of incident AF in patients with diabetes. Strict BP control should be emphasized for these patients.

**Supplementary Information:**

The online version contains supplementary material available at 10.1186/s12933-023-01736-4.

## Introduction

One in 11 adults has diabetes mellitus (DM) globally, and this population group is expected to rise to 700 million by 2045 [1, 2]. Deaths due to DM have doubled since 1990 [3]. Cardiovascular disease is estimated to account for one-third of DM deaths, primarily due to coronary artery disease and stroke [4]. Thus, managing cardiovascular risk factors is essential in reducing the mortality and morbidity associated with DM.

Among patients with DM, the presence of hypertension or atrial fibrillation (AF) is associated with an increased risk of complications, including stroke [5, 6]. Furthermore, the population with DM exhibits a higher risk of AF when compared to that without DM [7, 8]. The combination of DM and hypertension has been associated with an up to three-fold increase in the prevalence of AF, compared to rates in people without DM [7]. One previous study proposed a predictive model for AF in patients with hypertension and DM with acceptable performance [9]. However, previous studies have primarily focused on the association between baseline hypertension and the incidence of AF [7–9]. The impact of accumulated hypertension burden on the risk of AF in patients with DM has not previously been explored.

In this study, we aimed to investigate the relationship between accumulated hypertension burden and incident AF in patients with DM using a large nationwide population-based cohort.

## Methods

This study utilized the nationwide claims database of the Korean National Health Insurance Service (NHIS). The NHIS covers the entire South Korean population. The NHIS database consists of demographic variables, mortality data, medical expenses, diagnoses encoded by the International Classification of Disease, Tenth Revision of Clinical Modification (ICD-10-CM), utilization of inpatient and outpatient services, and prescription records [10]. Furthermore, the National Health Screening Program for chronic diseases targets people over the age of 19 and includes data on physical examinations, laboratory results, chest radiographs, and self-reported questionnaires [11].

This study was conducted in accordance with the Declaration of Helsinki. The data were anonymized, and thus, the study was exempted from the Institutional Review Board (IRB) review of Seoul National University Hospital (IRB no. E-2204-040-1314). In addition, because the data from the NHIS were de-identified, obtaining informed consent was not feasible. The use of the NHIS database from 2009 to 2012 was authorized in 2022.

### Study population

An overview of the patient selection flow is depicted in Additional file 1: Figure S1. Patients with DM who underwent a National Health Insurance Corporation health examination between January 1, 2009, and December 31, 2012, were screened for the study (n = 2,746,078). Patients aged < 40 years (n = 191,249), and those with prevalent AF before enrollment were excluded. Patients who underwent three consecutive biannual health examinations, including the index health examination, were included (n = 550,044).

### Definition of accumulated hypertension burden

During the health examination, a trained clinician measured the patient’s brachial blood pressure (BP) with a sphygmomanometer or an oscillometer with an appropriate-sized cuff, with the patient in the sitting position, after at least 5 min of rest [12, 13]. The BP measured at each health examination was classified into four categories: ‘no hypertension’ (systolic blood pressure (SBP) < 130 mmHg and diastolic blood pressure (DBP) < 80 mmHg); stage 1 hypertension (SBP 130–139 mmHg and DBP 80–89 mmHg); stage 2 hypertension (SBP 140–159 mmHg and 90–99 mmHg); and stage 3 hypertension (SBP ≥ 160 mmHg or DBP ≥ 100 mmHg), consistent with previous hypertension guidelines [14, 15]. We used the basic hypertension definitions from the 2017 ACC guideline for high BP and divided stage 2 hypertension into 2 groups: stage 2 (SBP 140–159 mmHg and 90–99 mmHg) and stage 3 (SBP ≥ 160 mmHg or DBP ≥ 100 mmHg) for further detailed evaluation of hypertension burden.

**Fig. 1:**
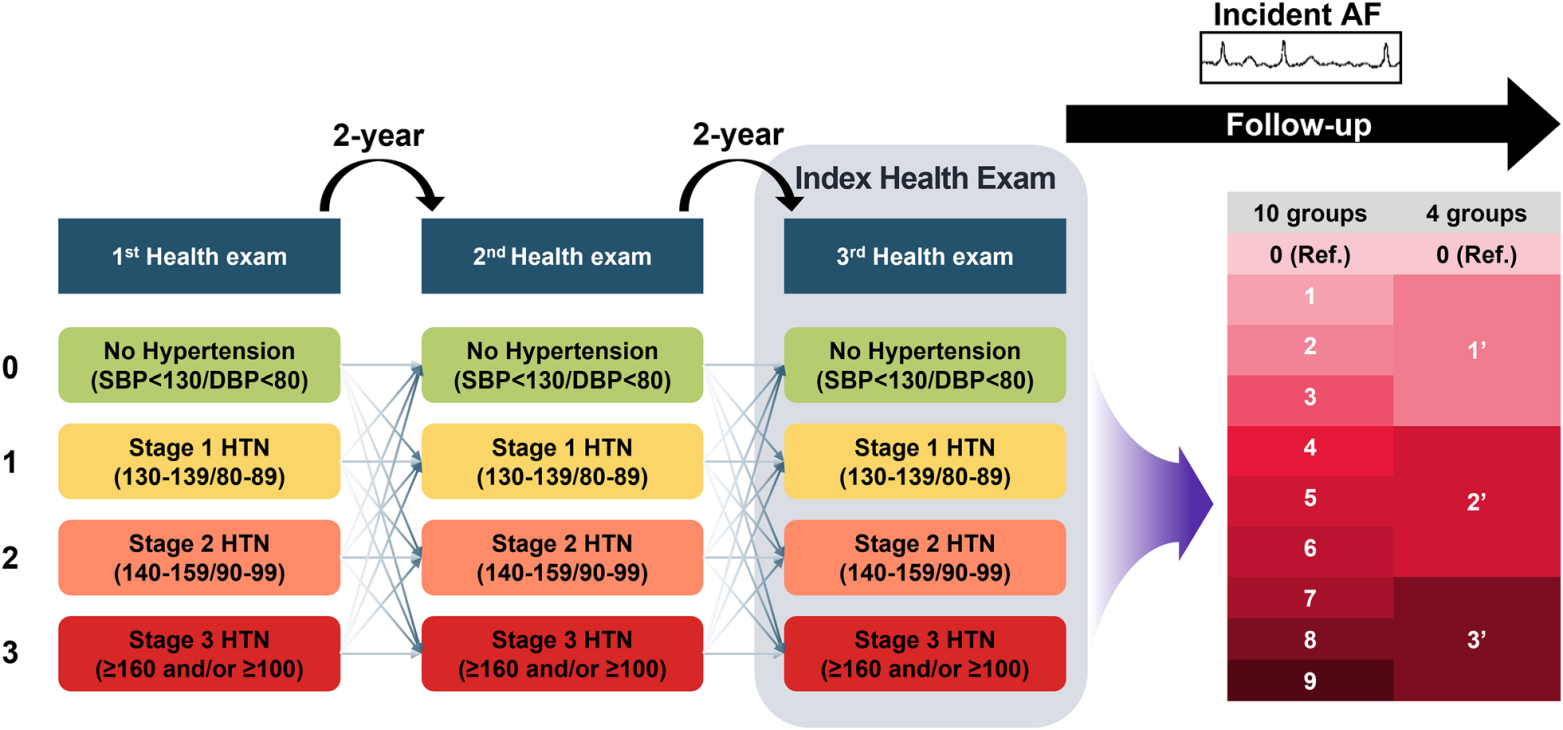
Study design. Abbreviation: AF, atrial fibrillation; BP, blood pressure; Exam, examination; Gr, grade; HTN, hypertension; Ref, reference

To quantify hypertension burden, we used a semiquantitative scoring system for the BP measured at each health examination: 0 points for no hypertension, 1 point for stage 1 hypertension, 2 points for stage 2 hypertension, and 3 points for stage 3 hypertension. To estimate the accumulation of hypertension status, the above grouping was applied to three consecutively performed health examinations, and the points from each health examination were summed for each subject. As a result, the patients were categorized into 10 groups based on hypertension burden (0–9) after three consecutive health examinations. Groups 1 to 9 were regrouped into three clusters: 1’ (1–3), 2’ (4–6), and 3’ (7–9), with group 0 as the reference group (Fig. 1). In additional statistical analysis, we selected subjects of SBP < 130 mmHg and DBP < 80 mmHg and assigned 0 point to normal BP (SBP < 120 mmHg and DBP < 80 mmHg) and 1 point to prehypertension (SBP < 130 mmHg and DBP < 80 mmHg). And the patients were categorized into 4 groups of 0–3.

### Covariates

Baseline demographic information, comorbidities defined by ICD-10-CM codes, prescribed drug use (anti-hypertensive medication and anti-diabetic medication), and laboratory results from the health examination are described in Table 1. Detailed definitions of inclusion and exclusion criteria (AF, hypertension, DM), comorbidities (chronic kidney disease [CKD], dyslipidemia, heart failure, myocardial infarction [MI], stroke, chronic obstructive pulmonary disease), health behavior (smoking, alcohol consumption, regular exercise), and household income are listed in Additional file 1: Table S1. Use of anti-hypertensive medications (thiazide, loop diuretics, aldosterone antagonists, alpha-/beta-blockers, calcium channel blockers, angiotensin-converting enzyme inhibitors, and angiotensin II receptor blockers were reviewed). Use of anti-diabetic medications (sulfonylureas, metformin, meglitinides, thiazolidinediones, dipeptidyl peptidase-4 inhibitors, α-glucosidase inhibitors, and insulin) were noted. All covariates were evaluated at the last (index, third) health examination, with comorbidities assessed a year prior to the index health examination. General health examination values of SBP, DBP, body mass index, and waist circumference were used. Laboratory results consisted of estimated glomerular filtration rate (eGFR), fasting glucose, total cholesterol, triglyceride, high-density lipoprotein cholesterol, and low-density lipoprotein cholesterol [16].

**Table 1:** Baseline characteristics of the study population according to hypertension burden group of 4

	Total (n = 514,967)	HTN burden				*p*-value
		0 (n = 49,812)	1’ (n = 260,938)	2’ (n = 173,256)	3’ (n = 30,961)	
Age, years						
Mean ± SD	61.3 ± 9.9	59.5 ± 9.3	60.7 ± 9.8	62.5 ± 9.8	62.5 ± 10.2	< .0001
< 65	61.1	70.0	63.7	55.9	54.2	
≥ 65	38. 9	30.0	36.3	44.1	45.8	
Sex (men)	59.6	52.4	60.1	60.4	61.5	< .0001
Comorbidities						
CKD	13.2	9.7	12.2	15.1	16.7	< .0001
Dyslipidemia	47.1	46.5	47.4	47.0	46.4	< .0001
Heart failure	1.6	1.3	1.5	1.6	1.9	< .0001
Prior MI	1.2	1.2	1.2	1.2	1.2	0.614
Prior stroke	5.6	4.0	5.3	6.4	6.7	< .0001
COPD	9.6	9.4	9.5	9.8	9.0	< .0001
Social history						
Smoking						< .0001
Non-smoker	59.1	61.1	57.7	60.2	61.2	
Ex-smoker	21.0	18.3	21.2	21.6	21.2	
Current smoker	19.9	20.6	21.2	18.2	17.6	
Alcohol consumption						< .0001
Non-drinker	61.6	69.5	62.2	59.5	56.0	
Mild to moderate (0–30 g/day)	30.6	26.6	30.7	31.3	32.6	
Heavy (≥ 30 g/day)	7.8	4.0	7.1	9.3	11.3	
Regular exercise	25.2	26.3	25.6	24.5	24.2	< .0001
Low income	20.7	19.1	20.5	21.2	22.1	< .0001
Medication						
HTN medication	56.9	27.1	49.4	72.5	81.6	< .0001
ACEi/ARB	47.0	25.6	41.8	57.9	64.3	< .0001
DM duration ≥ 5 years	60.5	65.3	61.1	59.0	55.2	< .0001
Insulin usage	12.0	14.0	12.2	11.3	10.5	< .0001
Oral anti-DM medication ≥ 3	24.8	26.4	25.6	23.7	21.0	< .0001
Metformin	70.6	73.1	71.8	69.2	63.5	< .0001
Sulfonylureas	69.1	66.3	68.8	70.6	67.9	< .0001
Meglitinides	2.7	3.2	2.7	2.5	2.1	< .0001
Alpha-glucosidase inhibitors	19.9	20.52	20.4	19.5	17.3	< .0001
Thiazolidinediones	10.8	12.4	11.3	10.0	8.5	< .0001
Dipeptidyl peptidase-4 inhibitors	12.6	15.9	13.6	10.8	8.8	< .0001
Health examination						
SBP (mmHg)	128.6 ± 15.3	112.4 ± 9.2	123.9 ± 11.2	136.4 ± 13.3	151.6 ± 15.4	< .0001
DBP (mmHg)	78.0 ± 9.8	67.8 ± 6.1	75.6 ± 7.8	82.3 ± 9.0	90.1 ± 10.7	< .0001
BMI (kg/m^2^)	24.8 ± 3.1	23.6 ± 2.8	24.7 ± 3.0	25.3 ± 3.2	25.6 ± 3.3	< .0001
WC (cm)	85.4 ± 8.1	81.9 ± 7.8	84.9 ± 7.9	86.7 ± 8.0	87.4 ± 8.3	< .0001
Laboratory results						
eGFR (mL/min/1.73 m2)	83.3 ± 35.3	85.7 ± 34.6	83.9 ± 35.5	82.1 ± 35.0	81.3 ± 35.9	< .0001
Fasting Glucose (mg/dL)	143.4 ± 48.1	141.6 ± 47.6	142.7 ± 47.9	143.8 ± 47.9	150.0 ± 50.9	< .0001
Total cholesterol (mg/dL)	187.4 ± 39.9	182.9 ± 38.5	186.1 ± 39.5	189.4 ± 40.3	194.6 ± 41.7	< .0001
HDL-C (mg/dL)	50.8 ± 20.4	51.1 ± 18.5	50.6 ± 19.7	50.9 ± 21.3	51.5 ± 21.5	< .0001
LDL-C (mg/dL)	105.6 ± 38.8	105.4 ± 37.6	105.2 ± 37.9	105.8 ± 39.9	108.1 ± 41.3	< .0001
*TG (mg/dL)	137.1 (136.9–137.3)	116.9 (116.4–117.5)	134.2 (133.9–134.5)	145.1 (144.7–145.5)	154.7 (153.8–155.7)	< .0001

Categorical variables were presented as a percentage and continuous variables were presented as mean and standard deviation

*ACEi* Angiotensin-converting enzyme inhibitors, *ARB* Angiotensin II Receptor Blockers, *BMI* body mass index, *CKD* chronic kidney disease, *COPD* chronic obstructive pulmonary disease, *DBP* diastolic blood pressure, *DM* diabetes mellitus, *eGFR* estimated glomerular filtration rate, *HDL-C* high density lipoprotein-cholesterol, *LDL-C* low density lipoprotein-cholesterol, *MI* myocardial infarction, *SBP* systolic blood pressure, *TG* triglyceride, *WC* waist circumference

^*^TG was presented as geometric mean (95% confidence interval)

### Study outcomes and follow-up

During the follow-up period, the incidence of AF was assessed as the primary outcome. AF was defined as the diagnosis of related ICD-10-CM codes (I48; AF and atrial flutter) for the first time during at least two different outpatient clinic visits or admissions or death [17]. The index date was the last (third) health examination. Patients were followed from the index date until the incident AF, disqualification from the NHIS (immigration or death), or the end of the study (December 31, 2018), whichever came first.

### Statistical analysis

For the baseline characteristics, continuous variables are presented as mean ± standard deviation (SD) and categorical variables as numbers and percentages. The comparison of baseline characteristics among different accumulated hypertension burden groups was performed with a linear trend test using a generalized linear model for continuous variables, the chi-square test, and the Cochran–Armitage trend test for categorical variables. The AF incidence rate (IR) was calculated by dividing the number of incident AF events by 1000 person-years at risk. For survival analysis, the Kaplan—Meier method and the log-rank test were used to determine the cumulative incidence of AF in relation to the accumulated hypertension burden. For multiple comparisons of Kaplan–Meier curve, Šidák correction was used. Cox proportional hazards regression models were used to evaluate the hazard ratio (HR) and 95% confidence interval (CI). Five stepwise Cox analysis models with adjustment for various combinations of covariates were performed as follows: (i) unadjusted model (model 1); (ii) model adjusted for age and sex (model 2); (iii) model adjusted for age, sex, comorbidities (CKD, dyslipidemia, heart failure, prior MI, prior stroke, smoking, alcohol consumption, regular exercise, and low income (model 3); (iv) model 3 with addition of DM, duration over 5 years, insulin usage, and more than three oral anti-diabetic medications (model 4); (v) model 4 with addition of SBP, fasting glucose, total cholesterol, and body mass index at the index health examination (model 5). The BP of the last health examination was adjusted in model 5 to adjust the effect of the most recent BP status.

Subgroup analyses were performed according to age (< 65 and ≥ 65 years), sex, the presence of CKD, prior MI or stroke, insulin usage, more than three oral anti-diabetic medications, thiazolidinediones, dipeptidyl peptidase-4 inhibitors, DM duration > 5 years, and anti-hypertensive medication, angiotensin-converting enzyme inhibitors (ACEi)/angiotensin II receptor blockers (ARB).

Statistical significance of p < 0.05 was used. All statistical analyses were performed using SAS version 9.4 (SAS Institute, Cary, North Carolina, USA).

## Results

### Study population

A total of 514,967 participants were included in the final study population. The patients were categorized into 10 groups and then regrouped into four clusters: 1′ (1–3), 2′ (4–6), and 3′ (7–9), with group 0 as the reference group. Of the whole cohort, the 10 groups of accumulated hypertension burden constituted 9.7% (n = 50,840), 14.2% (n = 74,963), 17.8% (n = 93,832), 18.7% (n = 98,354), 15.2% (n = 79,871), 11.3% (n = 59,612), 7.1% (n = 37,157), 3.9% (n = 20,370), 1.6% (n = 8,374), and 0.6% (n = 3,011) of patients, respectively. Baseline characteristics according to the four clusters are described in Table 1, and those in the 10 groups are described in Additional file 1: Table S2.

### Hypertension burden clusters

In the four clusters, participants in the higher accumulated hypertension burden group were older, but the prevalence of comorbidities did not show a linear trend. A higher accumulated hypertension burden was associated with heavy alcohol consumption, less regular exercise, and a lower income. Those with a higher accumulated hypertension burden were also more likely to receive anti-hypertensive medications, although prescription of oral anti-diabetic medications or insulin and duration of DM > 5 years were less common. In addition, the higher accumulated hypertension burden group had higher mean BP, body mass index, and waist circumference at the index health examination. Laboratory results showed lower eGFR and higher fasting glucose, total cholesterol, and triglyceride levels in the clusters with higher hypertension burden.

### Risk of incident AF according to accumulated hypertension burden

**Fig. 2:**
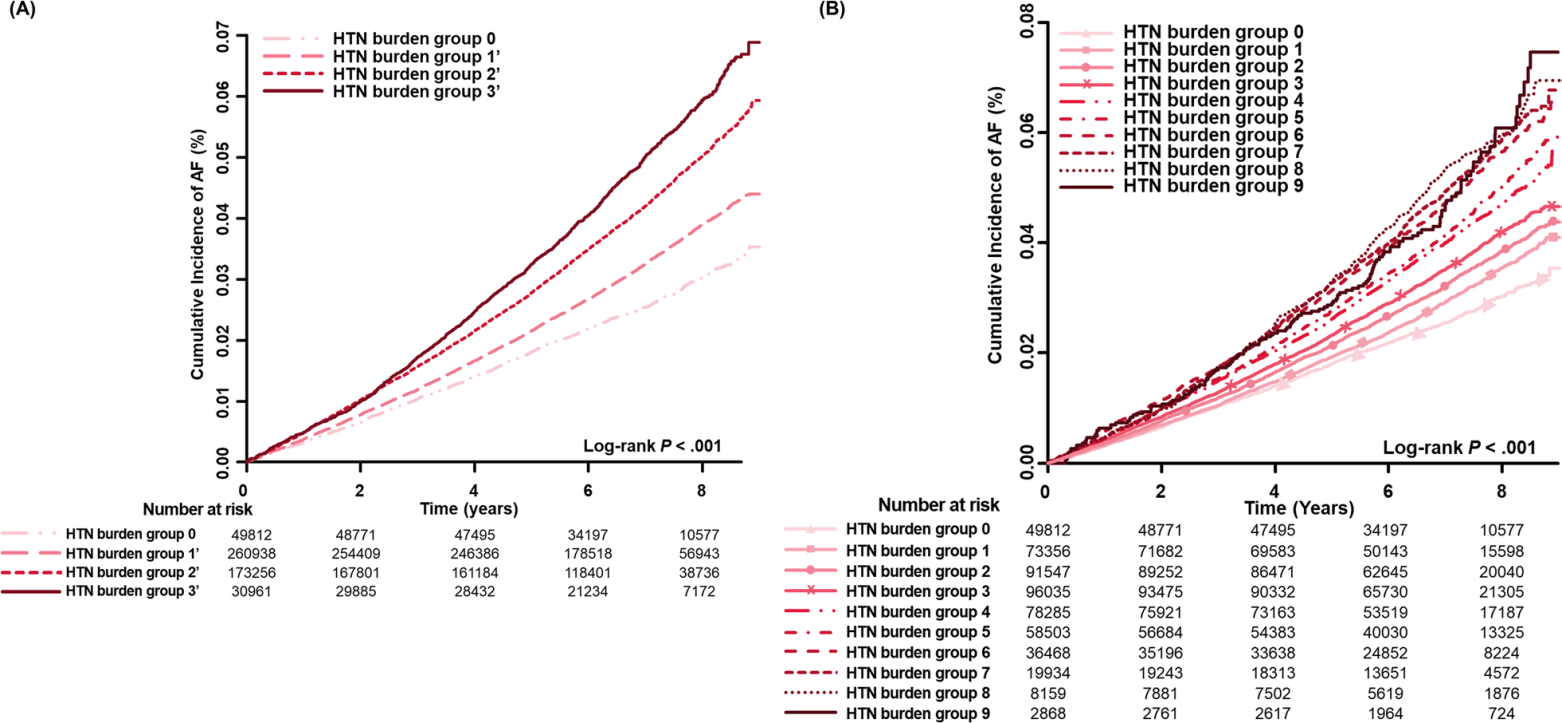
Cumulative incidence curves of AF stratified by hypertension burden; **A** group of 10 and **B** group of 4. Abbreviation: AF, atrial fibrillation; HTN, hypertension

During a mean follow-up duration of 6.7 (SD 1.7) years, AF was newly diagnosed in 18,561 patients (3.5% of the total population; incidence rate of 5.3 per 1,000 person-years). Both IR and HR increased with increasing accumulated hypertension burden (Additional file 1: Tables S3 and S4, respectively). The cumulative incidence curves for AF according to the hypertension burden are shown in Fig. 2. Compared with patients with a hypertension burden of 0, those with a hypertension burden of 1 or higher showed a higher risk of AF.

**Fig. 3:**
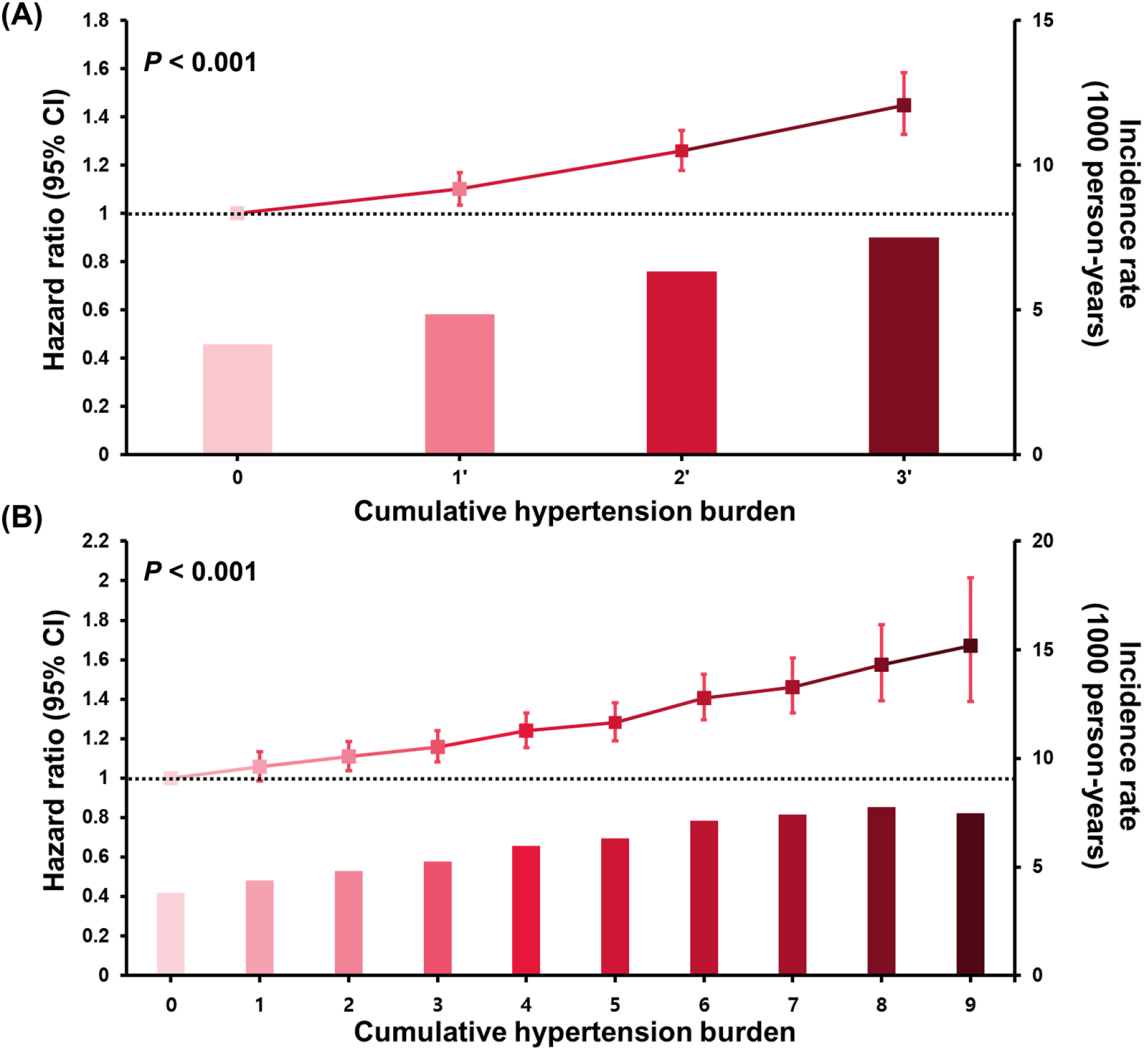
Association between cumulative hypertension burden and incident AF in diabetic subjects; **A** group of 10 **B** group of 4. Abbreviation: AF, atrial fibrillation; CI, confidence interval

Increased AF risk was seen in accumulated hypertension burden in the ten groups, as follows: 6%, 11%, 16%, 24%, 28%, 41%, 46%, 57%, and 67%, respectively (*P* < 0.001). When the study population was divided into four clusters according to hypertension burden (hypertension burden 0, 1 to 3 [group 1′], 4 to 6 [group 2′], and 7 to 10 [group 3′]), increased AF risk was observed by 10%, 26%, and 45% in groups 1′, 2′, and 3′, respectively, compared to those with hypertension burden 0 (*P* < 0.001). The associations between the accumulated hypertension burden and the risk of incident AF by adjusted HR (Model 5) are presented in Fig. 3.

Among the subjects with SBP < 130 mmHg and DBP < 80 mmHg, those who had prehypertension also showed an increased risk of AF compared to those who sustained normal BP (P = 0.0019) (Additional file 1: Table S5). Those who had BP at a range of prehypertension more than twice showed a similar risk to those who had prehypertension all the time (Additional file 1: Table S5, Figure S2).

### Subgroup analysis

The results of subgroup analyses are presented in Table 2. AF incidence was higher in the subgroups of age > 65 years, CKD, prior MI or stroke, insulin use, DM duration > 5 years, and use of anti-hypertensive medication. The subgroup of patients with three or more oral anti-diabetic medications and insulin, considered to have more advanced DM, was consistent with the main results. The severity of DM, as presumed by the prescription of more than three oral anti-diabetic medications or insulin, did not show a significant interaction. The prescription of specific anti-diabetic medication (thiazolidinediones or dipeptidyl peptidase-4 inhibitors) and anti-hypertensive medication (ACEi/ARB) did not affect the risk of AF.

**Table 2:** Subgroup analyses according to hypertension burden group of 4

Subgroup	HTN burden	Number	AF			
			Event	IR per 1000 PY	Adjusted HR*	*p* for interaction
Age						
< 65	0	34,889	565	2.38	1 (Reference)	0.116
	1′	166,256	3336	2.94	1.13 (1.03–1.23)	
	2′	96,778	2509	3.80	1.34 (1.22–1.48)	
	3′	16,778	494	4.33	1.54 (1.35–1.75)	
≥ 65	0	14,923	706	7.42	1 (Reference)	
	1′	94,682	5096	8.43	1.08 (0.99–1.17)	
	2′	76,478	4751	9.74	1.20 (1.11–1.31)	
	3′	14,183	1038	11.53	1.39 (1.25–1.54)	
Sex						
Male	0	26,098	795	4.62	1 (Reference)	0.223
	1′	156,937	5319	5.13	1.07 (0.99–1.15)	
	2′	104,696	4501	6.55	1.22 (1.13–1.32)	
	3′	19,031	912	7.33	1.36 (1.23–1.51)	
Female	0	23,714	476	2.96	1 (Reference)	
	1′	104,001	3113	4.44	1.16 (1.05–1.28)	
	2′	68,560	2759	5.99	1.33 (1.19–1.47)	
	3′	11,930	620	7.78	1.60 (1.41–1.82)	
CKD						
No	0	45,003	1080	3.59	1 (Reference)	0.099
	1′	229,093	6663	4.35	1.07 (1.00- 1.14)	
	2′	147,161	5585	5.69	1.23 (1.15–1.32)	
	3′	25,805	1139	6.63	1.41 (1.29–1.55)	
Yes	0	4809	191	6.04	1 (Reference)	
	1′	31,845	1769	8.58	1.29 (1.11–1.50)	
	2′	26,095	1675	10.07	1.42 (1.22–1.65)	
	3′	5156	393	12.16	1.66 (1.39–1.98)	
Prior MI or stroke						
No	0	47,243	1151	3.64	1 (Reference)	0.799
	1′	244,447	7510	4.60	1.10 (1.03–1.17)	
	2′	160,328	6408	6.01	1.26 (1.17–1.35)	
	3′	28,568	1355	7.16	1.46 (1.33–1.60)	
Yes	0	2569	120	7.39	1 (Reference)	
	1′	16,491	922	8.80	1.13 (0.93–1.37)	
	2′	12,928	852	10.45	1.27 (1.04–1.54)	
	3′	2393	177	11.73	1.38 (1.09–1.75)	
Insulin usage						
No	0	42,828	1035	3.60	1 (Reference)	0.573
	1′	229,158	6941	4.53	1.08 (1.01–1.16)	
	2′	153,770	6109	5.96	1.25 (1.16–1.34)	
	3′	27,697	1298	7.06	1.44 (1.31–1.58)	
Yes	0	6984	236	5.16	1 (Reference)	
	1′	31,780	1491	7.25	1.19 (1.04–1.36)	
	2′	19,486	1151	9.29	1.31 (1.14–1.52)	
	3′	3264	234	11.43	1.51 (1.25–1.82)	
Oral anti-diabetic medication ≥ 3						
No	0	36,672	924	3.78	1 (Reference)	0.422
	1′	194,051	6048	4.68	1.08 (1.00–1.15)	
	2′	132,145	5417	6.19	1.25 (1.16–1.34)	
	3′	24,458	1178	7.30	1.44 (1.30–1.58)	
Yes	0	13,140	347	3.93	1 (Reference)	
	1′	66,887	2384	5.33	1.17 (1.04–1.31)	
	2′	41,111	1843	6.73	1.29 (1.15–1.45)	
	3′	6503	354	8.27	1.48 (1.27–1.73)	
Thiazolidinediones						
No	0	43,627	1125	3.88	1 (Reference)	0.300
	1′	231,529	7430	4.84	1.09(1.02 1.16)	
	2′	155,954	6544	6.36	1.25 (1.17–1.34)	
	3′	28,323	1392	7.47	1.44(1.31–1.58	
Yes	0	6185	146	3.39	1 (Reference)	
	1′	29,409	1002	4.94	1.22 (1.02–1.45)	
	2′	17,302	716	6.04	1.28 (1.07–1.54)	
	3′	2638	140	7.87	1.54 (1.22–1.95)	
Dipeptidyl peptidase-4 inhibitors						
No	0	41,899	1112	3.92	1 (Reference)	0.652
	1′	225,592	7488	4.92	1.09 (1.02–1.17)	
	2′	154,525	6634	6.42	1.26 (1.17–1.35)	
	3′	28,237	1421	7.57	1.45 (1.32–1.59)	
Yes	0	7913	159	3.25	1 (Reference)	
	1′	35,346	944	4.35	1.16 (0.98–1.37)	
	2′	18,731	626	5.48	1.25 (1.05–1.49)	
	3′	2724	111	6.75	1.44 (1.13–1.85)	
DM duration ≥ 5 years						
No	0	17,283	362	3.15	1 (Reference)	0.057
	1′	101,438	2599	3.86	1.04 (0.93–1.16)	
	2′	71,014	2449	5.19	1.21 (1.08–1.35)	
	3′	13,879	505	5.48	1.26 (1.10–1.46)	
Yes	0	32,529	909	4.17	1 (Reference)	
	1′	159,500	5833	5.48	1.13 (1.05–1.21)	
	2′	102,242	4811	7.11	1.28 (1.18–1.38)	
	3′	17,082	1027	9.17	1.55 (1.40–1.71)	
Anti-hypertensive medication						
No	0	36,331	758	3.09	1 (Reference)	0.950
	1′	132,152	3156	3.53	1.05 (0.97–1.14)	
	2′	47,629	1368	4.24	1.15 (1.05–1.26)	
	3′	5701	172	4.47	1.27 (1.07–1.51)	
Yes	0	13,481	513	5.85	1 (Reference)	
	1′	128,786	5276	6.26	1.06 (0.97–1.16)	
	2′	125,627	5892	7.14	1.17 (1.06–1.29)	
	3′	25,260	1360	8.20	1.33 (1.19–1.49)	
ACEi/ARB						
No	0	37,079	799	3.20	1 (Reference)	0.807
	1′	151,812	4009	3.91	1.08 (1.00–1.17)	
	2′	72,916	2567	5.21	1.24 (1.14–1.35)	
	3′	11,043	447	5.98	1.40 (1.23–1.58)	
Yes	0	12,733	472	5.70	1 (Reference)	
	1′	109,126	4423	6.21	1.08 (0.98–1.19)	
	2’	100,340	4693	7.16	1.21 (1.09–1.33)	
	3′	19,918	1085	8.38	1.39 (1.24–1.56)	

*ACEi Angiotensin-converting enzyme inhibitors, ARB Angiotensin II Receptor Blockers, AF* atrial fibrillation, *CKD* chronic kidney disease, *DM* diabetes mellitus, *HR* hazard ratio, *HTN* hypertension, *IR* incidence rate, *MI* myocardial infarction, *PY* person-year

^*^Adjusted HR: Model 5 (adjustment of age, sex, CKD, dyslipidemia, heart failure, prior MI, prior stroke, smoking, alcohol, regular exercise, low income, DM duration over 5 years, insulin usage, more than 3 oral antidiabetic medications, SBP, fasting glucose, total cholesterol, and BMI at latest (index) health examination)

## Discussion

In this study, our principal findings were as follows: (1) patients with DM with a higher accumulated hypertension burden had an increased risk of incident AF, and (2) accumulated hypertension burden showed a positive correlation with the risk of AF in a population with DM, regardless of the severity of DM. To the best of our knowledge, this is the first study to evaluate the risk of incident AF in patients with DM and an accumulated hypertension burden.

DM is one of the most common chronic medical conditions, affecting one in 11 adults globally [1]. Patients with DM are at a higher risk of major cardiovascular adverse events and mortality compared to people without DM [18]. People with DM are more likely to develop AF by atrial structural remodeling and adrenergic activation and have an even higher risk of major coronary events, strokes, heart failure, and mortality when present in combination with AF [19–21]. DM patients with AF may also experience increased AF symptom burden and a lower quality of life [22]. Because cumulative exposure to DM status itself increases the risk of AF by 3% for each additional year [23, 24], it is important to control other modifiable risk factors of AF in patients with DM.

Hypertension is a common modifiable risk factor that affects the pathogenesis, management, and prognosis of AF [25]. Hypertension is responsible for more than one-fifth of all incident AF and shows a linear increase in risk when the exposure is accumulated [12, 26]. Hypertension affects more than two-thirds of patients with DM [27], and the coexistence of hypertension in patients with DM increases the risk of AF three-fold [7]. However, the latter study was a cross-sectional observational study that focused on the presence or absence of baseline hypertension [7]. The accumulated effect of hypertension on AF development in patients with DM has not been previously evaluated.

Although the pathophysiology of AF remains under investigation, there are possible explanations for the association between hypertension and AF. In animal models, hypertension is associated with atrial remodeling, especially fibrosis, and higher AF inducibility [25, 28]. Long-term exposure to hypertension is also associated with left ventricular hypertrophy, leading to increased left atrial pressure and subsequent atrial enlargement [29, 30]. Such structural remodeling leads to an increased incidence of AF in a dose-dependent response to cumulative hypertension burden, as shown in our study and by others [26]. As such, a change in left ventricular hypertrophy can be prevented or even improved with intensive BP control and anti-hypertensive medications [31, 32], and strict BP control should lower the incidence of AF in patients with DM.

In the subgroup analyses, the patients with anti-hypertensive medication had a higher incidence of AF but the incidence was similar to those without anti-hypertensive medication, unlike the previous study conducted on the general population [26]. This difference could be caused by the effect of DM outweighing hypertension on the incidence of AF [7]. Another interesting result in the subgroup analyses was that the severity of DM, as determined by insulin usage [33], did not show a significant interaction with AF risk. Despite the increased absolute AF incidence in the insulin group (as was seen in previous study [34]), the accumulated hypertension burden had a similar impact on the risk of AF in patients with DM regardless of insulin usage. Thus, strict BP control is important in all patients with DM, irrespective of the severity of DM.

In this study, the accumulated hypertension burden persistently showed an increased AF risk regardless of the known duration of DM. Accumulated DM burden is known to be associated with an increased AF incidence [23]; therefore, a long-term comprehensive treatment plan for the evaluation and management of DM and hypertension is needed to lower AF risk in patients with longer DM duration. This is aligned with the current approach to characterization and evaluation of patients with AF [35], followed by a holistic or integrated care approach to AF management [36]. Such integrated care management has been associated with improved clinical outcomes [37] and is recommended in guidelines [38].

### Study limitations

This study had several limitations. First, we used I48 to define AF. The use of ICD-10-CM codes in AF diagnosis may be less accurate than reviewing the actual electrocardiogram. However, the AF definition using I48 was previously validated using 628 patients with a positive predictive value as high as 94.1% [39]. There still is a possibility of underestimation of the actual AF incidence and surveillance bias on the contrary as well. Second, although this study used a health examination provided by the Korean National Health Insurance Cooperation, which covers at least 74% of the adults in Korea [40], the number of subjects with diabetes who went through three consecutive biannual health examinations was limited. Thus, the possibility of selection bias was inevitable in the current study design. Third, the Korean National Health Insurance Corporation health examination does not include data on 24-h BP or medication compliance. Different effects on the risk of AF expected in subjects with white-coat hypertension, uncontrolled hypertension, or difficult-to-control hypertension cannot be discriminated in this study. Fourth, the effect of novel anti-diabetic drugs such as sodium-glucose transporter 2 inhibitors (SGLT2i) and glucagon-like peptide-1 receptor agonists (GLP1a) could not be assessed as Korea started prescribing SGLT2i after year 2015, and the number of GLP1a prescription was too low. Fifth, the BP change during the follow-up period was not identified, and thus its effect might have been underestimated. Lastly, we studied the Korean population, which is considered homogeneous; hence, generalizability to other multi-ethnic populations is limited.

## Conclusion

Among patients with DM, accumulated hypertension burden was associated with an increased risk of incident AF. Strict BP control should be emphasized in managing patients with DM to help reduce the risk of AF-related complications in this population.

## Supplementary Information

**Additional file 1: Table S1. **Definitions of covariates. **Table S2. **Baseline characteristics of the study population according to hypertension burden group of 10. **Table S3. **Hazard ratios for atrial fibrillation according to the hypertension burden group of 4. **Table S4. **Hazard ratios for atrial fibrillation according to the hypertension burden group of 10. **Table S5.** Hazard ratios for atrial fibrillation among subjects with SBP <130 mmHg and DBP < 80 mmHg. **Figure S1.** Overview of the patient flow. **Figure S2.** Cumulative incidence curves of AF among subjects with SBP <130 mmHg and DBP < 80 mmHg.

## Data Availability

The data that support the findings of this study are available from the corresponding author upon reasonable request.

## Abbreviations

AF: Atrial fibrillation
BP: Blood pressure
CKD: Chronic kidney disease
CI: Confidence interval
DBP: Diastolic blood pressure
DM: Diabetes mellitus
eGFR: Estimated glomerular filtration rate
HR: Hazard ratio
ICD-10-CM: International Classification of Disease, Tenth Revision of Clinical Modification
IR: Incidence rate
MI: Myocardial infarction
NHIS: Korean National Health Insurance Service
SBP: Systolic blood pressure
SD: Standard deviation
