# Supplementary material for: Accumulated hypertension burden on atrial fibrillation risk in diabetes mellitus: a nationwide population study
Source: Cardiovasc Diabetol. 2023 Jan 19;22:12. doi: 10.1186/s12933-023-01736-4 (PMC9854085; doi:10.1186/s12933-023-01736-4)
Supplement: Supplementary file 1 — Additional file 1: Table S1. Definitions of covariates. Table S2. Baseline characteristics of the study population according to hypertension burden group of 10. Table S3. Hazard ratios for atrial fibrillation according to the hypertension burden group of 4. Table S4. Hazard ratios for atrial fibrillation according to the hypertension burden group of 10. Table S5. Hazard ratios for atrial fibrillation among subjects with SBP <130 mmHg and DBP < 80 mmHg. Figure S1. Overview of the patient flow. Figure S2. Cumulative incidence curves of AF among subjects with SBP <130 mmHg and DBP < 80 mmHg. [file 12933_2023_1736_MOESM1_ESM.docx]

Additional file 1: Material

**Additional file 1: Tables**

**Table S1.** Definitions of covariates.

**Table S2.** Baseline characteristics of the study population according to hypertension burden group of 10

**Table S3.** Hazard ratios for atrial fibrillation according to the hypertension burden group of 4

**Table S4.** Hazard ratios for atrial fibrillation according to the hypertension burden group of 10

**Table S5.** Hazard ratios for atrial fibrillation among subjects with SBP <130 mmHg and DBP < 80 mmHg

**Additional file 1: Figures**

**Figure S1.** Overview of the patient flow.

**Figure S2.** Cumulative incidence curves of AF among subjects with SBP <130 mmHg and DBP < 80 mmHg

**Additional file 1: Tables**

**Additional file 1: Table S1. Definitions of covariates.**

| **Diagnosis** | **ICD-10-CM code and definition** | **Diagnostic definition** |
| --- | --- | --- |
| **Inclusion/exclusion criteria** |  |  |
| **Atrial fibrillation** | I48.0-48.4, I48.9 | Admission or outpatient department≥1 |
| **Hypertension** | I10-I13, I15; and minimum one prescription of anti-hypertensive medication (thiazide, loop diuretic, aldosterone antagonist, alpha-/beta-blocker, calcium channel blocker, angiotensin-converting enzyme inhibitor, and angiotensin II receptor blocker). | Admission≥1 or outpatient department≥2 |
|  | or systolic/diastolic blood pressure ≥ 140/90 mmHg | Index health examination |
| **Diabetes mellitus** | E11-E14; and minimum one prescription of anti-diabetic drugs (sulfonylurea, metformin, meglitinide, thiazolidinedione, dipeptidyl peptidase-4 inhibitor, α-glucosidase inhibitor, and insulin). | Admission≥1 or outpatient department≥1 |
|  | Or fasting glucose level ≥ 126 mg/dL | Index health examination |
| **Comorbidities** |  |  |
| **Chronic kidney disease** | Estimated glomerular filtration rate <60 ml/min/1.73m^2^ | Index health examination |
| **Chronic obstructive pulmonary disease** | J41-44 | Admission or outpatient department≥1 |
| **Dyslipidemia** | E78 | Admission or outpatient department≥1 |
|  | Or Total cholesterol ≥ 240 mg/dL | Index health examination |
| **Heart failure** | I50 | Admission or outpatient department≥1 |
| **Prior MI** | I21, I22 | Admission or outpatient department≥1 |
| **Prior stroke** | I63, I64 | Admission or outpatient department≥1 |
| **Health exam questionnaire** |  |  |
| **Smoking**  **Ex**  **Current** | Ex-smoker at the 1^st^ examination and sustaining non-smoking till the 2^nd^ examination  Current smoker at the 2^nd^ examination regardless of the smoking status at the 1^st^ examination. | Index health examination |
| **Alcohol consumption**  **Mild to moderate**  **Heavy** | Alcohol consumption >0g to <30g per day  Alcohol consumption ≥30g per day | Index health examination |
| **Regular exercise** | Performing a moderate physical activity more than 30 minutes at least 5 times per week or strenuous physical activity more than 20 minutes at least 3 times per week. | Index health examination |
| **Low income** | Income lowest 20% among the entire Korean population and supported by the medical aid | Index health examination |

Abbreviations: ICD, international classification of disease; CM, clinical modification.

**Additional file 1: Table S2. Baseline characteristics of the study population according to hypertension burden group of 10.**

|  | **Total**  **(n= 514967)** | **HTN burden** | | | | | | | | | | ***p*-value** |
| --- | --- | --- | --- | --- | --- | --- | --- | --- | --- | --- | --- | --- |
|  |  | **0**  **(n=49812)** | **1**  **(n= 73356)** | **2**  **(n=91547)** | **3**  **(n=96035)** | **4**  **(n=78285)** | **5**  **(n=58503)** | **6**  **(n=36468)** | **7**  **(n=19934)** | **8**  **(n=8159)** | **9**  **(n=2868)** |  |
| **Age, years** |  |  |  |  |  |  |  |  |  |  |  |  |
| **Mean ± SD** | 61.3 ± 9.9 | 59.5 ± 9.3 | 60.1 ± 9.6 | 60.7 ± 9.8 | 61.1 ± 9.9 | 62.2 ± 9.8 | 62.6 ± 9.8 | 63.0 ± 9.8 | 62.8 ± 10.0 | 62.4 ± 10.3 | 60.9 ± 10.6 | <.0001 |
| **<65** | 61.1 | 70.0 | 66.4 | 63.9 | 61.5 | 57.5 | 55.4 | 53.2 | 53.3 | 54.5 | 59.5 |  |
| **≥65** | 38.9 | 30.0 | 33.6 | 36.1 | 38.5 | 42.6 | 44.6 | 46.8 | 46.7 | 45.5 | 40.5 |  |
| **Sex (men)** | 59.6 | 52.4 | 57.5 | 60.3 | 62.0 | 60.9 | 60.1 | 60.0 | 61.1 | 61.2 | 64.9 |  |
| **Comorbidities** |  |  |  |  |  |  |  |  |  |  |  |  |
| **CKD** | 13.2 | 9.7 | 10.9 | 12.2 | 13.2 | 14.3 | 15.4 | 16.3 | 16.4 | 17.4 | 16.5 | <.0001 |
| **Dyslipidemia** | 47.1 | 46.5 | 47.6 | 47.3 | 47.3 | 47.2 | 47.0 | 46.7 | 46.3 | 46.8 | 45.5 | 0.0009 |
| **Heart failure** | 1.6 | 1.3 | 1.4 | 1.5 | 1.5 | 1.6 | 1.6 | 1.8 | 2.0 | 2.0 | 1.6 | <.0001 |
| **Prior MI** | 1.2 | 1.2 | 1.2 | 1.2 | 1.2 | 1.2 | 1.2 | 1.3 | 1.1 | 1.2 | 1.0 | 0.8651 |
| **Prior ischemic stroke** | 5.6 | 4.0 | 4.8 | 5.4 | 5.6 | 6.3 | 6.4 | 6.8 | 6.9 | 6.8 | 5.7 | <.0001 |
| **COPD** | 9.56 | 9.4 | 9.5 | 9.6 | 9.5 | 9.8 | 9.9 | 9.4 | 9.3 | 9.0 | 7.0 | <.0001 |
| **Social history** |  |  |  |  |  |  |  |  |  |  |  |  |
| **Smoking** |  |  |  |  |  |  |  |  |  |  |  | <.0001 |
| **Non-smoker** | 59.1 | 61.1 | 58.3 | 57.6 | 57.2 | 59.3 | 60.7 | 61.4 | 61.6 | 61.4 | 58.4 |  |
| **Ex-smoker** | 21.0 | 18.3 | 20.2 | 21.2 | 22.0 | 21.8 | 21.3 | 21.4 | 21.2 | 21.4 | 20.7 |  |
| **Current smoker** | 19.9 | 20.6 | 21.5 | 21.2 | 20.8 | 18.9 | 18.0 | 17.1 | 17.2 | 17.3 | 20.9 |  |
| **Alcohol consumption** |  |  |  |  |  |  |  |  |  |  |  | <.0001 |
| **Non-drinker** | 61.6 | 69.5 | 65.2 | 62.3 | 59.9 | 59.8 | 59.3 | 59.0 | 56.8 | 55.7 | 51.9 |  |
| **Mild to moderate (0–30 g/day)** | 30.6 | 26.6 | 29.1 | 30.7 | 32.0 | 31.3 | 31.2 | 31.2 | 32.1 | 33.0 | 34.9 |  |
| **Heavy (≥30g/day)** | 7.8 | 4.0 | 5.7 | 7.1 | 8.1 | 8.9 | 9.6 | 9.9 | 11.1 | 11.3 | 13.3 |  |
| **Regular exercise** | 25.2 | 26.3 | 25.6 | 25.7 | 25.6 | 24.8 | 24.6 | 24.0 | 24.4 | 23.7 | 23.7 | <.0001 |
| **Low income** | 20.7 | 19.1 | 20.3 | 20.3 | 20.7 | 21.2 | 21.1 | 21.4 | 21.7 | 22.4 | 23.7 | <.0001 |
| **Medication** |  |  |  |  |  |  |  |  |  |  |  |  |
| **Antihypertensive medication** | 56.9 | 27.1 | 38.3 | 48.9 | 58.3 | 68.0 | 74.4 | 79.3 | 81.7 | 82.3 | 78.8 | <.0001 |
| **ACEi / ARB** | 47.0 | 25.6 | 34.0 | 41.5 | 48.1 | 54.7 | 59.2 | 62.8 | 64.6 | 65.0 | 60.7 | <.0001 |
| **DM duration ≥ 5 years** | 60.5 | 65.3 | 63.1 | 61.1 | 59.7 | 59.6 | 58.8 | 58.0 | 56.5 | 54.5 | 48.1 | <.0001 |
| **Insulin usage** | 12.0 | 14.0 | 13.2 | 12.2 | 11.4 | 11.4 | 11.3 | 11.0 | 10.7 | 10.7 | 9.1 | <.0001 |
| **Oral antidiabetic medication ≥ 3** | 24.8 | 26.4 | 26.5 | 25.8 | 24.8 | 24.2 | 23.7 | 22.8 | 21.4 | 21.4 | 17.4 | <.0001 |
| **Metformin** | 70.6 | 73.1 | 73.0 | 71.9 | 70.9 | 70.2 | 68.9 | 67.4 | 65.0 | 63.0 | 54.4 | <.0001 |
| **Sulfonylureas** | 69.1 | 66.3 | 68.1 | 68.9 | 69.3 | 70.5 | 70.7 | 70.5 | 68.9 | 68.2 | 59.8 | <.0001 |
| **Meglitinides** | 2.7 | 3.2 | 2.9 | 2.7 | 2.6 | 2.6 | 2.4 | 2.3 | 2.2 | 2.0 | 2.0 | <.0001 |
| **Alpha-glucosidase inhibitors** | 19.9 | 20.5 | 21.0 | 20.5 | 19.9 | 19.7 | 19.7 | 18.9 | 17.7 | 17.5 | 14.5 | <.0001 |
| **Thiazolidinediones** | 10.8 | 12.4 | 12.0 | 11.2 | 10.8 | 10.3 | 10.0 | 9.4 | 8.9 | 8.3 | 6.8 | <.0001 |
| **Dipeptidyl peptidase-4 inhibitors** | 12.6 | 15.9 | 14.6 | 13.7 | 12.6 | 11.6 | 10.3 | 10.0 | 9.1 | 8.7 | 7.2 | <.0001 |
| **Health examination** |  |  |  |  |  |  |  |  |  |  |  |  |
| **SBP (mmHg)** | 128.6 ± 15.3 | 112.4 ± 9.2 | 118.6 ± 10.1 | 123.5 ± 10.5 | 128.2 ± 10.9 | 132.8 ± 12.0 | 137.4 ± 13.0 | 142.6 ± 13.8 | 148.3 ± 14.7 | 155.1 ± 14.0 | 165.2 ± 14.1 | <.0001 |
| **DBP (mmHg)** | 78.0 ± 9.8 | 67.8 ± 6.1 | 72.2 ± 7.1 | 75.4 ± 7.5 | 78.3 ± 7.6 | 80.5 ± 8.2 | 82.9 ± 8.9 | 85.3 ± 9.5 | 88.4 ± 10.2 | 91.9 ± 10.4 | 97.4 ± 11.4 | <.0001 |
| **BMI (kg/m^2^)** | 24.8 ± 3.1 | 23.6 ± 2.8 | 24.2 ± 2.9 | 24.6 ± 3.0 | 25.0± 3.0 | 25.2 ± 3.1 | 25.3 ± 3.2 | 25.5 ± 3.2 | 25.6 ± 3.3 | 25.6 ± 3.3 | 25.7 ± 3.4 | <.0001 |
| **WC (cm)** | 85.4 ± 8.1 | 81.9 ± 7.8 | 83.7 ± 7.8 | 84.9 ± 7.8 | 85.8 ± 7.9 | 86.4 ± 8.0 | 86.8 ± 8.1 | 87.1 ± 8.2 | 87.4 ± 8.3 | 87.4 ± 8.4 | 87.5 ± 8.4 | <.0001 |
| **Laboratory results** |  |  |  |  |  |  |  |  |  |  |  |  |
| **eGFR (mL/min/1.73 m2)** | 83.3 ± 35.3 | 85.7 ± 34.6 | 84.7 ± 35.5 | 83.9 ± 35.1 | 83.3 ± 36.0 | 82.5 ± 34.6 | 82.0 ± 35.2 | 81.5 ± 35.6 | 81.4 ± 35.9 | 80.5 ± 29.5 | 82.6 ± 49.4 | <.0001 |
| **Fasting Glucose (mg/dL)** | 143.4 ± 48.1 | 141.6 ± 47.6 | 142.3 ± 47.9 | 142.7 ± 48.2 | 142.9 ± 47.7 | 143.0 ± 47.6 | 144.0 ± 48.0 | 145.0 ± 48.2 | 148.7 ± 50.1 | 150.5 ± 51.4 | 157.6 ± 53.8 | <.0001 |
| **Total cholesterol (mg/dL)** | 187.4 ± 39.9 | 182.9 ± 38.5 | 184.5 ± 39.2 | 186.1 ± 39.4 | 187.2 ± 39.8 | 188.5 ± 40.0 | 189.8 ± 40.6 | 190.8 ± 40.6 | 193.3 ± 41.2 | 195.9 ± 42.3 | 200.0 ± 42.7 | <.0001 |
| **HDL-C (mg/dL)** | 50.8 ± 20.2 | 51.1 ± 18.5 | 50.7 ± 19.5 | 50.5 ± 19.9 | 50.5 ± 19.6 | 50.7 ± 20.4 | 51.0 ± 20.9 | 51.3 ± 23.6 | 51.4 ± 21.0 | 51.9 ± 23.8 | 51.7 ± 17.8 | <.0001 |
| **LDL-C (mg/dL)** | 105.6 ± 38.8 | 105.4 ± 37.6 | 105.1 ± 37.0 | 105.2 ± 37.6 | 105.1 ± 38.8 | 105.6 ± 40.2 | 105.9 ± 39.9 | 106.1 ± 39.4 | 107.4 ± 40.3 | 108.9 ± 42.8 | 110.6 ± 43.2 | <.0001 |
| ***TG (mg/dL)** | 137.1 (136.9-137.3) | 116.9 (116.4-117.5) | 127.1 (126.6-127.6) | 134.4 (133.9-134.8) | 139.7 (139.2-140.2) | 142.8 (142.3-143.4) | 146.2 (145.5-146.8) | 148.5 (147.6-149.3) | 152.7 (151.6-153.9) | 156.3 (154.4-158.2) | 164.4 (161.1-167.8) | <.0001 |

Categorical variables were presented as a percentage and continuous variables were presented as mean and standard deviation.
*TG was presented as geometric mean (95% confidence interval).
Abbreviation: ACEi, Angiotensin-converting enzyme inhibitors; ARB, Angiotensin II Receptor Blockers; BMI, body mass index; CKD, chronic kidney disease; COPD, chronic obstructive pulmonary disease; DBP, diastolic blood pressure; DM, diabetes mellitus; eGFR, estimated glomerular filtration rate; HDL-C, high density lipoprotein-cholesterol; LDL-C, low density lipoprotein-cholesterol; MI, myocardial infarction; SBP, systolic blood pressure; TG, triglyceride; WC, waist circumference.

**Table S3.** Hazard ratios for atrial fibrillation according to the hypertension burden group of 4.

| **HTN burden** | **Number** | **Event** | **IR** | **Model 1**  **HR (95% CI)** | **Model 2**  **HR (95% CI)** | **Model 3**  **HR (95% CI)** | **Model 4**  **HR (95% CI)** | **Model 5**  **HR (95% CI)** |
| --- | --- | --- | --- | --- | --- | --- | --- | --- |
| **0** | 49812 | 1271 | 3.82 | 1 (Reference) | 1 (Reference) | 1 (Reference) | 1 (Reference) | 1 (Reference) |
| **1’** | 260938 | 8432 | 4.85 | 1.27 (1.20 -1.35) | 1.14 (1.07 -1.21) | 1.13 (1.06 -1.19) | 1.14 (1.07 -1.21) | 1.10 (1.04 -1.17) |
| **2’** | 173256 | 7260 | 6.32 | 1.65 (1.56 -1.75) | 1.33 (1.25 -1.41) | 1.30 (1.23 -1.39) | 1.33 (1.25 -1.41) | 1.26 (1.18 -1.35) |
| **3’** | 30961 | 1532 | 7.50 | 1.96 (1.82 -2.11) | 1.55 (1.44 -1.67) | 1.51 (1.41 -1.63) | 1.55 (1.44 -1.67) | 1.45 (1.33 -1.58) |
| ***P* - value** | | | | <0.001 | <0.001 | <0.001 | <0.001 | <0.001 |

IR is presented per 1000PY.

Multivariable adjusted model included age, sex, CKD, dyslipidemia, heart failure, prior MI, prior stroke, smoking, alcohol, regular exercise, low income, DM duration over 5 years, insulin usage, more than 3 oral antidiabetic medications, SBP, fasting glucose, total cholesterol, and BMI at latest (index) health examination.

Model 1: unadjusted

Model 2: age and sex

Model3: Age, Sex, CKD, dyslipidemia, heart failure, prior MI, prior stroke, smoking, alcohol, regular exercise, low income

Model4: model 3 + DM duration over 5 years, insulin usage, more than 3 oral antidiabetic medications

Model5: model 4 + SBP, fasting glucose, total cholesterol, BMI at last (index) health examination

Abbreviations: AF, atrial fibrillation; BMI, body mass index; CI, confidence interval; CKD, chronic kidney disease; DM, diabetes mellitus; HR, hazard ratio; IR, incidence rate; MI, myocardial infarction; PY, person-years; SBP, systolic blood pressure.

**Table S4.** Hazard ratios for atrial fibrillation according to the hypertension burden group of 10.

| **HTN burden** | **Number** | **Event** | **IR** | **Model 1**  **HR (95% CI)** | **Model 2**  **HR (95% CI)** | **Model 3**  **HR (95% CI)** | **Model 4**  **HR (95% CI)** | **Model 5**  **HR (95% CI)** |
| --- | --- | --- | --- | --- | --- | --- | --- | --- |
| **0** | 49812 | 1271 | 3.82 | 1 (Reference) | 1 (Reference) | 1 (Reference) | 1 (Reference) | 1 (Reference) |
| **1** | 73356 | 2136 | 4.37 | 1.14 (1.07 - 1.23) | 1.07 (1.00 - 1.15) | 1.07 (1.00 - 1.14) | 1.07 (1.00 - 1.15) | 1.06 (0.99 - 1.13) |
| **2** | 91547 | 2945 | 4.83 | 1.26 (1.18 - 1.35) | 1.14 (1.06 - 1.21) | 1.12 (1.05 - 1.20) | 1.13 (1.06 - 1.21) | 1.11 (1.04 - 1.19) |
| **3** | 96035 | 3351 | 5.24 | 1.37 (1.29 - 1.46) | 1.19 (1.12 - 1.27) | 1.17 (1.10 - 1.25) | 1.19 (1.12 - 1.27) | 1.16 (1.08 - 1.24) |
| **4** | 78285 | 3094 | 5.96 | 1.56 (1.46 - 1.66) | 1.28 (1.19 - 1.36) | 1.26 (1.18 - 1.34) | 1.28 (1.20 - 1.37) | 1.24 (1.16 - 1.33) |
| **5** | 58503 | 2451 | 6.32 | 1.65 (1.54 - 1.77) | 1.32 (1.23 - 1.41) | 1.30 (1.21 - 1.39) | 1.32 (1.23 - 1.41) | 1.28 (1.19 - 1.38) |
| **6** | 36468 | 1715 | 7.12 | 1.86 (1.73 – 2.00) | 1.45 (1.35 - 1.56) | 1.42 (1.32 - 1.53) | 1.45 (1.35 - 1.56) | 1.41 (1.30 - 1.53) |
| **7** | 19934 | 973 | 7.40 | 1.93 (1.78 - 2.10) | 1.51 (1.39 - 1.64) | 1.47 (1.36 - 1.60) | 1.51 (1.39 - 1.64) | 1.46 (1.33 - 1.61) |
| **8** | 8159 | 418 | 7.76 | 2.03 (1.82 - 2.27) | 1.62 (1.45 - 1.81) | 1.58 (1.41 - 1.76) | 1.61 (1.44 - 1.80) | 1.57 (1.39 - 1.78) |
| **9** | 2868 | 141 | 7.47 | 1.95 (1.64 - 2.32) | 1.68 (1.41 - 2.00) | 1.64 (1.38 - 1.95) | 1.70 (1.43 - 2.02) | 1.67 (1.39 - 2.01) |
| ***P* - value** | | | | <0.001 | <0.001 | <0.001 | <0.001 | <0.001 |

IR is presented per 1000PY.

Multivariable adjusted model included age, sex, CKD, dyslipidemia, heart failure, prior MI, prior stroke, smoking, alcohol, regular exercise, low income, DM duration over 5 years, insulin usage, more than 3 oral antidiabetic medications, SBP, fasting glucose, total cholesterol, and BMI at latest (index) health examination.

Model 1: unadjusted

Model 2: age and sex

Model3: Age, Sex, CKD, dyslipidemia, heart failure, prior MI, prior stroke, smoking, alcohol, regular exercise, low income

Model4: model 3 + DM duration over 5 years, insulin usage, more than 3 oral antidiabetic medications

Model5: model 4 + SBP, fasting glucose, total cholesterol, BMI at last (index) health examination

Abbreviations: AF, atrial fibrillation; BMI, body mass index; CI, confidence interval; CKD, chronic kidney disease; DM, diabetes mellitus; HR, hazard ratio; IR, incidence rate; MI, myocardial infarction; PY, person-years; SBP, systolic blood pressure.

**Table S5.** Hazard ratios for atrial fibrillation among subjects with SBP <130 mmHg and DBP < 80 mmHg

| **HTN burden** | **Number** | **Event** | **IR** | **Model1**  **HR (95% CI)** | **Model2**  **HR (95% CI)** | **Model3**  **HR (95% CI)** | **Model4**  **HR (95% CI)** | **Model5**  **HR (95% CI)** |
| --- | --- | --- | --- | --- | --- | --- | --- | --- |
| **0** | 22792 | 522 | 3.41 | 1 (Reference) | 1 (Reference) | 1 (Reference) | 1 (Reference) | 1 (Reference) |
| **1’’** | 45951 | 1343 | 4.39 | 1.29 (1.16 - 1.42) | 1.12 (1.01 - 1.24) | 1.10 (0.99 - 1.22) | 1.11 (1.00 - 1.22) | 1.11 (1.00 - 1.23) |
| **2’’** | 71083 | 2501 | 5.29 | 1.55 (1.41 - 1.70) | 1.21 (1.10 - 1.33) | 1.18 (1.07 - 1.29) | 1.19 (1.08 - 1.31) | 1.20 (1.09 - 1.32) |
| **3’’** | 40836 | 1435 | 5.31 | 1.56 (1.41 - 1.72) | 1.12 (1.02 - 1.24) | 1.10 (0.99 - 1.22) | 1.11 (1.01 - 1.23) | 1.20 (1.07 - 1.35) |
| P-value | | | | <.0001 | 0.0005 | 0.0034 | 0.0021 | 0.0019 |

IR is presented per 1000PY.

Multivariable adjusted model included age, sex, CKD, dyslipidemia, heart failure, prior MI, prior stroke, smoking, alcohol, regular exercise, low income, DM duration over 5 years, insulin usage, more than 3 oral antidiabetic medications, SBP, fasting glucose, total cholesterol, and BMI at latest (index) health examination.

Model 1: unadjusted

Model 2: age and sex

Model3: Age, Sex, CKD, dyslipidemia, heart failure, prior MI, prior stroke, smoking, alcohol, regular exercise, low income

Model4: model 3 + DM duration over 5 years, insulin usage, more than 3 oral antidiabetic medications

Model5: model 4 + SBP, fasting glucose, total cholesterol, BMI at last (index) health examination

Abbreviations: AF, atrial fibrillation; BMI, body mass index; CI, confidence interval; CKD, chronic kidney disease; DM, diabetes mellitus; HR, hazard ratio; IR, incidence rate; MI, myocardial infarction; PY, person-years; SBP, systolic blood pressure.

**Additional file 1: Figures**

**Figure S1.** Overview of the patient flow.

**
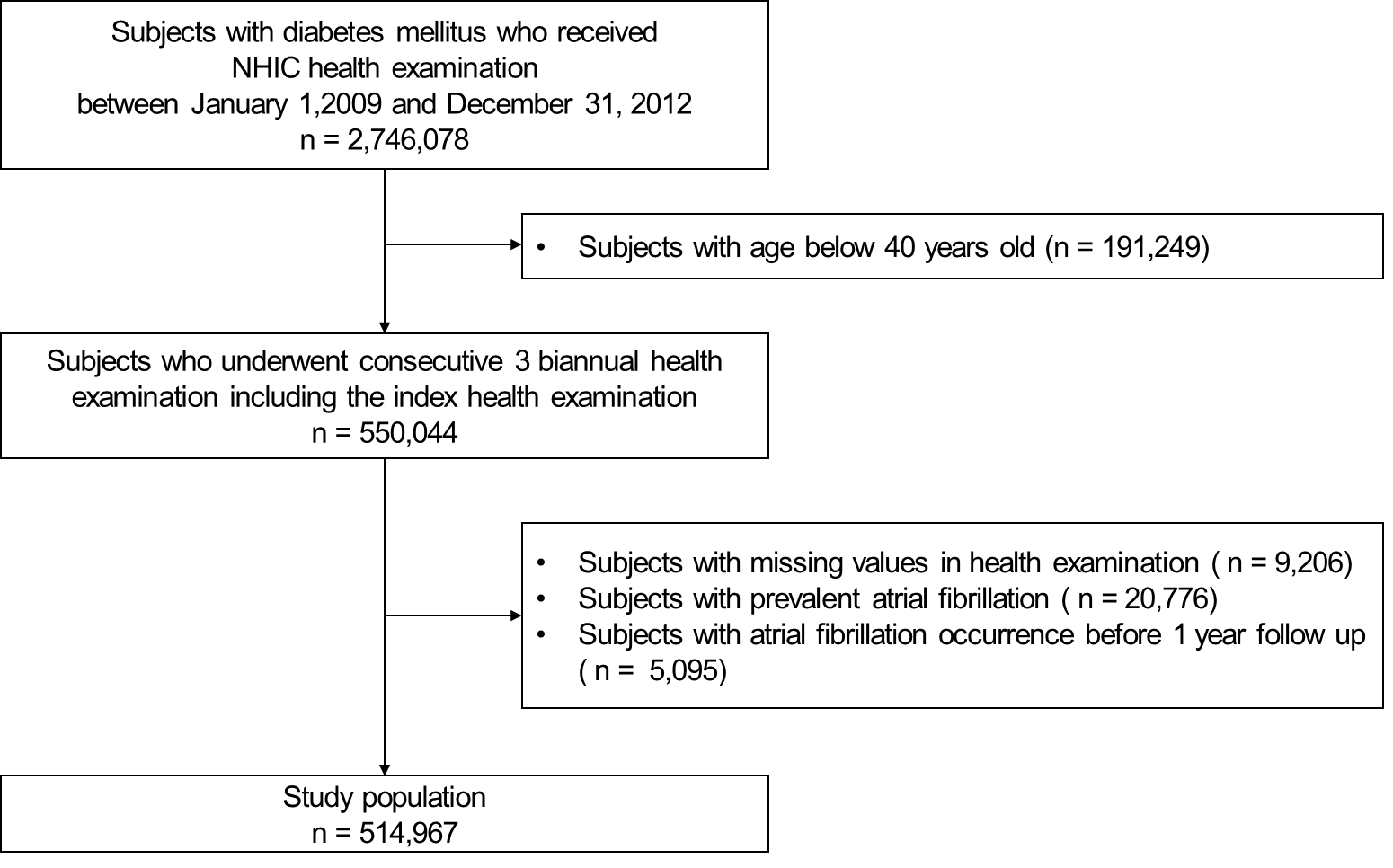
**

Abbreviations: NHIC, National health insurance corporation

**Figure S2.** Cumulative incidence curves of AF among subjects with SBP <130 mmHg and DBP < 80 mmHg


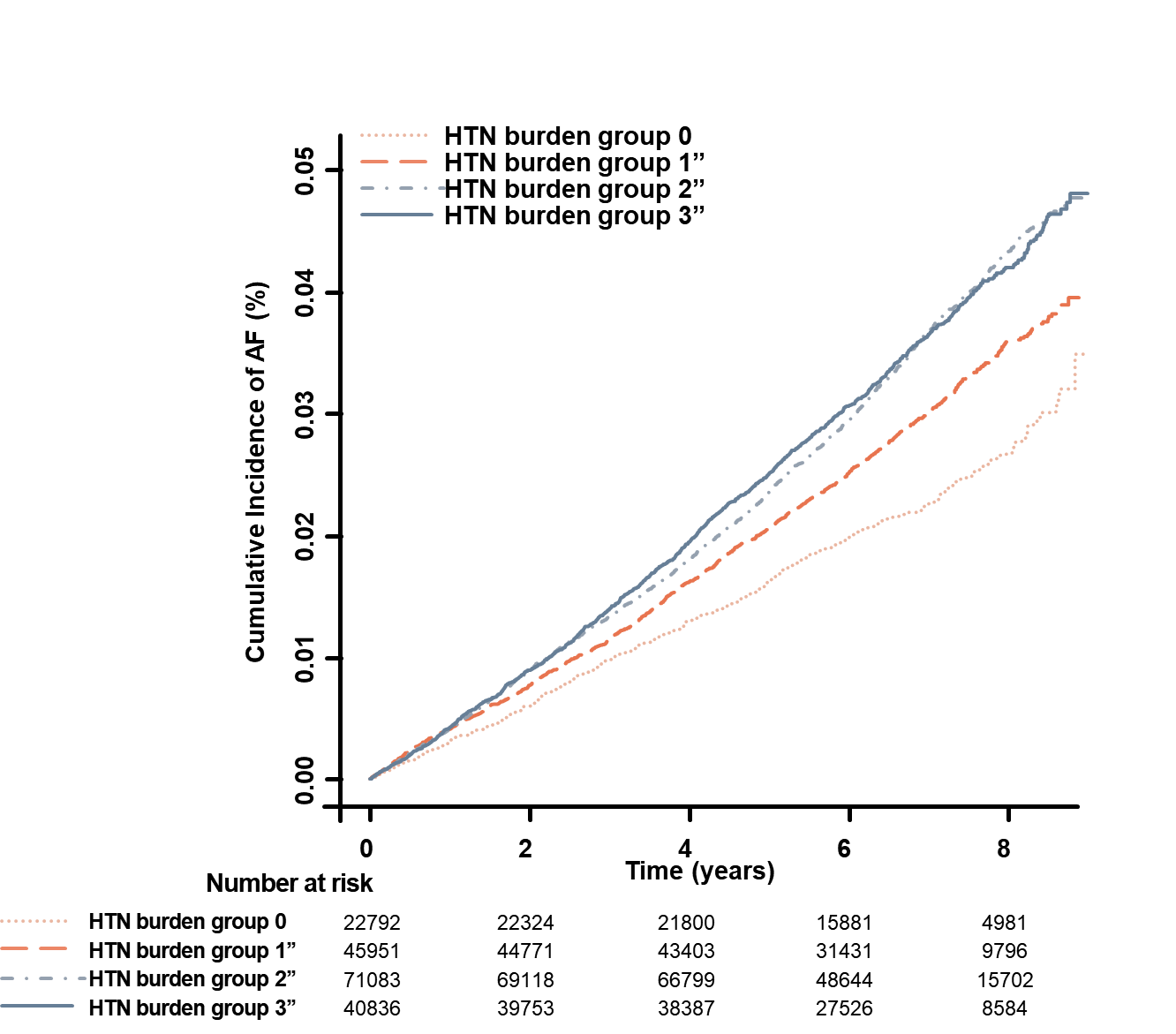


Abbreviation: AF, atrial fibrillation HTN, hypertension.
